# Pharmacokinetics of neomycin in plasma, urine, and feces of donkeys (*Equus asinus*) after a single intragastric administration

**DOI:** 10.3389/fvets.2026.1800606

**Published:** 2026-03-11

**Authors:** Honglei Qu, Yuhan Wei, Shijie Liu, Boying Dong, Yulong Feng, Shimeng Huang, Lihong Zhao, Bowen Yang, Qiugang Ma

**Affiliations:** 1 National Engineering Research Center for Gelatin-Based Traditional Chinese Medicine, Dong-E-E-Jiao Co., Ltd., Liaocheng, China; 2 College of Animal Science and Technology, Hebei Agricultural University, Baoding, China; 3 State Key Laboratory of Animal Nutrition, College of Animal Science and Technology, China Agricultural University, Beijing, China

**Keywords:** antibiotics, donkey, metabolism, neomycin, pharmacokinetics

## Abstract

Neomycin is an aminoglycoside antibiotic widely used in veterinary medicine for the treatment of gastrointestinal infections. However, pharmacokinetic studies of neomycin in donkeys are limited. The present study aimed to investigate the pharmacokinetic profiles of neomycin in donkey plasma, urine, and feces following a single intragastric administration, and to evaluate its suitability for clinical use in donkeys. A total of five healthy male donkeys with similar body weights were selected and administered a single dose of 30 mg·kg^−1^ body weight (BW) neomycin by gavage. The concentrations of neomycin in plasma, urine, and feces were determined. The results showed that neomycin was rapidly absorbed in donkeys, with a T_max_ of 0.85 ± 0.36 h and a C_max_ of 4.05 ± 1.99 μg·mL^−1^ in plasma. The elimination half-life (T_₁/₂*λ*_) was 32.14 ± 12.71 h, indicating a slow elimination rate. The cumulative excretion of neomycin in urine accounted for 15.08% of the administered dose, while fecal excretion accounted for 70.99%, suggesting low systemic absorption following intragastric administration. In conclusion, the low systemic absorption and high fecal excretion of intragastric neomycin in donkeys justify its use for intestinal infections. Given the prolonged elimination half-life of the absorbed drug, its use should be restricted to local intestinal therapy to minimize systemic exposure and toxicity risks. This study contributes to the advancement of precision medicine in donkey internal medicine, offering an evidence-based foundation for optimizing therapeutic strategies and minimizing systemic risks in donkeys.

## 1 Introduction

Donkeys fulfill varied roles across the globe, including used as draft and companion animals as well as sources of high-value commodities (1, 2). In China, products such as *Colla Corii Asini* (Ejiao) (3), donkey meat (4), and donkey milk (5) hold significant commercial importance. Despite their economic value, traditional rearing systems, particularly in regions with limited resources, frequently subject donkeys to poor environmental conditions and increased susceptibility to bacterial infections. These challenges contribute to substantial health issues and economic losses.

Effective treatment of these health issues depends on understanding how drugs are metabolized in donkeys, a key question in pharmacology. However, there is a notable lack of pharmacokinetic (PK) studies in donkeys, leading to the common practice of extrapolating dosages from equine data (6, 7). This approach carries substantial risk given documented interspecies differences in physiological and metabolic profiles, such as disparities in body water compartments and the activity of cytochrome P450 enzymes (8, 9). The resulting knowledge gap may contribute to inadequate drug concentrations or toxic effects, which could further exacerbate the challenge of antimicrobial resistance. Therefore, studying how drugs move through donkeys’ bodies is an urgent priority. This knowledge is essential to ensure treatments work properly, protect the animals’ health, and support the long-term viability of donkey farming. The present study selected neomycin, a commonly used antimicrobial agent in clinical practice, to investigate its pharmacokinetics in donkeys following intragastric administration.

**Figure 1:**
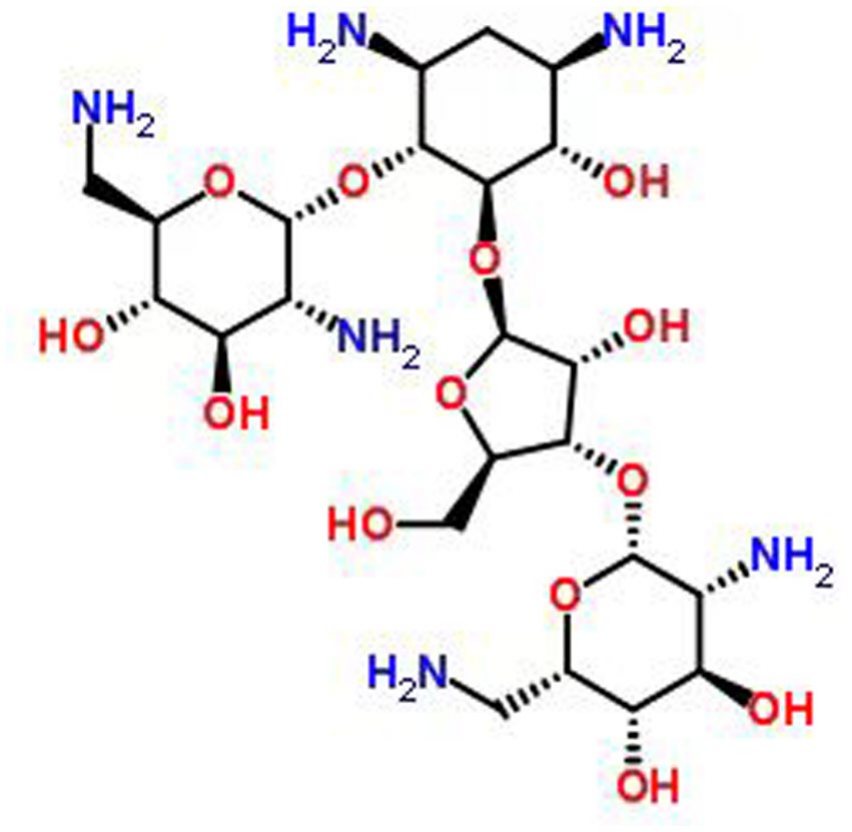
Chemical formula of neomycin.

Neomycin is a natural antibiotic produced by the actinomycete *Streptomyces fradiae* and belongs to the aminoglycoside class. Its chemical structure consists of aminosugars linked to aminocyclitol by an oxygen bridge (10–12). The chemical structure of neomycin is illustrated in Figure 1. Neomycin is clinically utilized primarily as a mixture of its active components, Neomycin B and C, in the form of neomycin sulfate (13–15). This broad-spectrum agent exerts its bactericidal effect by binding to the bacterial 30S ribosomal subunit, thereby inhibiting protein synthesis (16). It demonstrates efficacy against a range of Gram-negative bacteria and is commonly employed in veterinary medicine for the treatment of gastrointestinal and other bacterial infections (17). However, irrational use of neomycin can cause toxic effects in animals, including ototoxicity, nephrotoxicity, and neurotoxicity (18–21). Pharmacokinetic studies of neomycin have been reported in horses (22, 23), Swine (24), sheep (25), cattle (26–28), and chickens (29), but data in donkeys remain notably absent for donkeys. Given the physiological and metabolic differences between donkeys and horses (8, 9), extrapolating dosage regimens from other species is scientifically unsound and clinically risky. Therefore, to directly address this critical knowledge gap and provide evidence-based guidance for clinical medication in donkey internal medicine, this study was designed as an applied pharmacokinetic investigation. It aimed to investigate the pharmacokinetic profiles of neomycin in plasma, urine, and feces of donkeys following intragastric administration, providing a data basis for the rational clinical use of neomycin in the treatment of bacterial infection in donkeys.

## 2 Materials and methods

### 2.1 Chemicals and reagents

Neomycin sulfate (98%) was purchased from China Animal Husbandry Industry Co., Ltd. (Nanjing, China). The neomycin standard solutions (purity ≥ 99.0%) was purchased from the Research and Monitoring Institute of Environmental Protection, Ministry of Agriculture, China. The isopropanol, acetonitrile, methanol, formic acid, and ammonium acetate were of chromatographically pure. Trichloroacetic acid, ammonia water, and potassium dihydrogen phosphate were of analytical grade.

### 2.2 Animals and management

Animal experiments were conducted in accordance with protocols approved by the Institutional Animal Care and Use Committee (IACUC) of China Agricultural University (grant No. Aw80803202-1-1). A total of five healthy 9-month-old male donkeys with an average body weight of 145.10 ± 8.11 kg were used in this study. After a 7-day trial period, donkeys were fasted overnight prior to the experiment. Other basic information is presented in Table 1. At 1 h before the trial, each donkey was guided into a metabolic cage (2.0 m × 0.8 m × 1.8 m). Throughout the experimental period, every donkey was fed with 2 kg of fattening concentrate feed every day, with free access to coarse feed (corn straw) and drink water.

**Table 1:** Basic information about experimental animals.

Items	Donkey number					x_ ± *sd*
	1	2	3	4	5	
Body weight (kg)	137.00	158.50	141.00	144.00	145.00	145.10 ± 7.26
Single administered dose (mg·kg^−1^)	30.00	30.00	30.00	30.00	30.00	–
Administered dose (mg)	4,110	4,755	4,230	4,320	4,350	4,353 ± 217.66
Concentrated feed intake (kg·d^−1^)	2.00	2.00	2.00	2.00	2.00	–
Coarse fodder intake (kg·d^−1^)	2.04	2.21	2.01	2.10	1.69	2.01 ± 0.17
Total feces volume (kg)	28.65	30.50	32.25	32.85	20.25	28.90 ± 4.56
Water intake (L·d^−1^)	38.33	31.67	63.33	68.89	29.44	46.33 ± 16.51
Total urine volume (L)	6.88	10.17	4.30	11.60	7.76	8.14 ± 2.55

### 2.3 Experimental design

Based on the study by Aschbacher and Feil (30) in horses, each donkey were intragastrically administrated a single dose of 30 mg·kg^−1^ BW neomycin by gavage. Neomycin sulfate was dissolved in sterile physiological saline (0.9% NaCl) to prepare a stock solution. The concentration was adjusted so that each animal received a consistent dose of 30 mg·kg^−1^ BW at a convenient gavage volume. The solution was freshly prepared on the day of administration and administered via oral gavage. After administration, blood samples were collected from the jugular vein and placed them in heparinized anticoagulant tubes. The blood samples were centrifuged at 1,500 × g for 20 min at 4 °C to obtain plasma. Blood sample time collection points were set at 0.00, 0.08, 0.25, 0.42, 0.58, 0.75, 1.00, 1.50, 2.00, 2.50, 3.00, 4.00, 5.00, 6.00, 8.00, 10.00, 12.00, 24.00, 36.00, 48.00, and 72.00 h after administration. Urine and fecal samples were collected every 6 h. For urine collection, male donkeys were fitted with a custom-made external urine collection device attached to the genital area, enabling total urine collection without contamination or discomfort to the animals. Fecal samples were collected via rectal sampling, where all feces present in the rectum were retrieved at 6-h intervals, pooled, and thoroughly mixed to obtain a representative sample for analysis. All plasma, fecal, and urine samples were kept at −80 °C until further analysis.

### 2.4 Sample preparation and extraction

The concentrations of neomycin in donkey plasma, urine, and feces were determined using an established method with modifications (31). For plasma and urine, 1 mL of sample was processed. For feces, 1 g of lyophilized sample was used. Briefly, the sample was placed into a 50 mL centrifuge tube. Then, 25 μL of a mixed standard working solution (1.0 μg·mL^−1^) and 20 mL of 5% trichloroacetic acid solution were added. The mixture was vortexed for 1 min, ultrasonicated for 20 min, and centrifuged at 8,000 × g for 5 min. The supernatant was transferred to a new tube and adjusted to pH 7.5 ± 0.2 using ammonia water.

For solid-phase extraction (SPE) cleanup, the extract was loaded onto an Oasis HLB cartridge (200 mg, 6 mL; Waters, Milford, MA, United States), which had been preconditioned sequentially with 5 mL of methanol and 5 mL of ultrapure water. After sample loading, the cartridge was washed with 3 mL of ultrapure water and 5 mL of 5% aqueous methanol. Neomycin was eluted with 5 mL of a mixture of formic acid, isopropanol, and 0.002 mol·L^−1^ ammonium acetate aqueous solution (10:5:85, v/v/v). The eluate was collected, dried under a gentle nitrogen stream at 40 °C, and reconstituted in 1 mL of the initial mobile phase.

To mitigate matrix effects and ensure the analyte response fell within the optimal range of the detector, the final reconstituted extract from all samples, including the calibration standards and quality control samples prepared in blank fecal matrix for method validation, was subjected to a 50-fold dilution with the initial mobile phase prior to LC–MS/MS analysis. The calibration curves were therefore constructed using concentrations that reflect this dilution. Consequently, all reported concentrations (including the LOD, LOQ, and pharmacokinetic results) have been mathematically corrected for this 50-fold dilution factor and are expressed relative to the original dry weight of the fecal sample (μg·g^−1^ or mg·kg^−1^ dry weight).

### 2.5 UPLC-MS/MS analysis

Chromatographic separation was performed on an ACQUITY UPLC BEH C18 column (100 mm × 2.1 mm, 1.7 μm; Waters, Milford, MA, United States) maintained at 40 °C. The mobile phase consisted of (A) 0.002 mol·L^−1^ ammonium acetate in water with 1% formic acid and (B) acetonitrile with 1% formic acid. A gradient elution program was shown in Table 2. The flow rate was 0.3 mL·min^−1^, and the injection volume was 5 μL.

**Table 2:** Gradient elution condition of mobile phase.

Time (min)	Mobile Phase A (%)	Mobile Phase B (%)	Flow rate (mL·min^−1^)
0	90	10	0.3
1	90	10	0.3
1.5	10	90	0.3
2.5	10	90	0.3
3.5	90	10	0.3
4	90	10	0.3

Mass spectrometric detection was carried out on an AB SCIEX QTRAP® 5,500 system (Sciex, Framingham, MA, United States) operated in positive electrospray ionization (ESI^+^) mode. The ion source parameters were optimized as follows: ion spray voltage, 3.50 kV; source temperature, 150 °C; desolvation temperature, 450 °C; curtain gas flow, 50 L·h^−1^; and nebulizer gas flow, 700 L·h^−1^. Neomycin was monitored using multiple reaction monitoring (MRM). The declustering potential was set at 40 V. The specific precursor-to-product ion transitions for neomycin were m·z^−1^ 615.3 → 161.2 and m·z^−1^ 615.3 → 293.2, with corresponding collision energies (CE) of 25 eV and 20 eV, respectively. The first transition was used for quantification, and the second for confirmation.

### 2.6 UPLC method validation

The analytical method was validated according to the acceptance criteria outlined in Chinese Pharmacopeia (2020 Edition, Guidelines 9,101). Calibration curves for neomycin were constructed inblank matrices (plasma, urine, and feces) over a concentration range of 50, 100, 200, 500, 700, and 1,000 ng·mL^−1^ (or ng·g^−1^) for feces. Linearity was evaluated using the correlation coefficient (*R*^2^) of the weighted least-squares regression. The instrument response for both standard and sample solutions was confirmed to be within this established linear range. Additionally, a single-point calibration (at 50 ng·mL^−1^) was performed for routine sample batch analysis. Method validation parameters, including accuracy (expressed as recovery), precision, limit of detection (LOD), and limit of quantification (LOQ), were determined for each matrix.

### 2.7 Data analysis

Pharmacokinetic analysis was performed using non-compartmental methods with the linear trapezoidal rule in Phoenix WinNonlin (Version 8.1; Certara, Raleigh, NC, United States). Key pharmacokinetic parameters were calculated with reference to those described in previous studies (32). The first-order rate constant associated with the terminal (log-linear) phase of the curve (λz) was estimated by linear regression of the terminal data points. The terminal elimination half-life (T_1/2λz_) was calculated by T_1/2λz_ = 0.693/λz. In the plasma model, the peak plasma concentration (C_max_) and times to reach peak concentration (T_max_) were obtaineddirectly from the observed concentration-timedata. The area under the plasma concentration-time curve from time zero to infinity (AUC_₀–∞_) and the mean residence time (MRT) was calculated using using standard non-compartmental algorithms within the software. For the urinary excretion model, the urinary excretion rate, cumulative amount excreted versus the midpoint of each urine collection interval, and the area under the rate curve (AURC_₀–∞_) were calculated with reference to previously reported methods (33). All data are presented as mean ± standard deviation (SD).

## 3 Results

### 3.1 Method validation

The analytical method for determining neomycin concentrations in plasma, urine, and feces was fully validated. Calibration curves showed excellent linearity over the range of 50 to 1,000 ng·mL^−1^ (for plasma and urine) or ng·g^−1^ (for feces, dry weight). The correlation coefficients (*R*^2^) were 0.9989, 0.9993, and 0.9956 for plasma, urine, and feces, respectively.

The limits of detection (LOD) and quantification (LOQ) were determined based on signal-to-noise ratios of 3 and 10, respectively, and were further verified by the precision and accuracy at the LOQ level. As summarized in Table 3, the LOD and LOQ were 13.70 and 45.60 ng·mL^−1^ for plasma, 5.20 and 17.30 ng·mL^−1^ for urine, and 27.40 and 91.20 ng·g^−1^ for feces. The higher values in feces reflect the increased matrix complexity.

**Table 3:** Method validation parameters for the determination of neomycin in donkey plasma, urine, and feces.

Matrix	Linear range (ng·mL^−1^ or ng·g^−1^)	*R* ^2^	Accuracy (recovery, %)	Precision (RSD, %)	LOD (ng·mL^−1^ or ng·g^−1^)	LOQ (ng·mL^−1^ or ng·g^−1^)
Plasma	50–1,000	0.9989	96.80	8.50	13.70	45.60
Urine	50–1,000	0.9993	102.15	7.20	5.20	17.30
Feces	50–1,000	0.9956	84.56	12.10	27.40	91.20

Accuracy (Recovery, %): Percentage recovery of neomycin from spiked samples; Precision (RSD, %): Intra-day precision expressed as relative standard deviation; LOD: Limit of detection (signal-to-noise ratio ≥ 3); LOQ: Limit of quantification (signal-to-noise ratio ≥ 10). Validation was performed using blank matrices spiked with neomycin across the concentration range of 50–1,000 ng·mL^−1^ (ng·g^−1^ for feces). The reported concentration ranges for method validation refer to the nominal concentrations in the original biological samples prior to the 50-fold dilution step applied during sample preparation.

The accuracy and precision of the method were evaluated by analyzing spiked samples at three concentration levels. The mean recoveries were 96.80, 102.15, and 84.56% for plasma, urine, and feces, respectively, all within the acceptable range of 80–120%. The precision, expressed as relative standard deviation (RSD), was less than 15% for all matrices at all levels (Table 1). These results demonstrate that the developed method is sensitive, accurate, and precise for the quantification of neomycin in the studied biological matrices.

### 3.2 Pharmacokinetic parameters of neomycin in the plasma of donkeys

**Figure 2:**
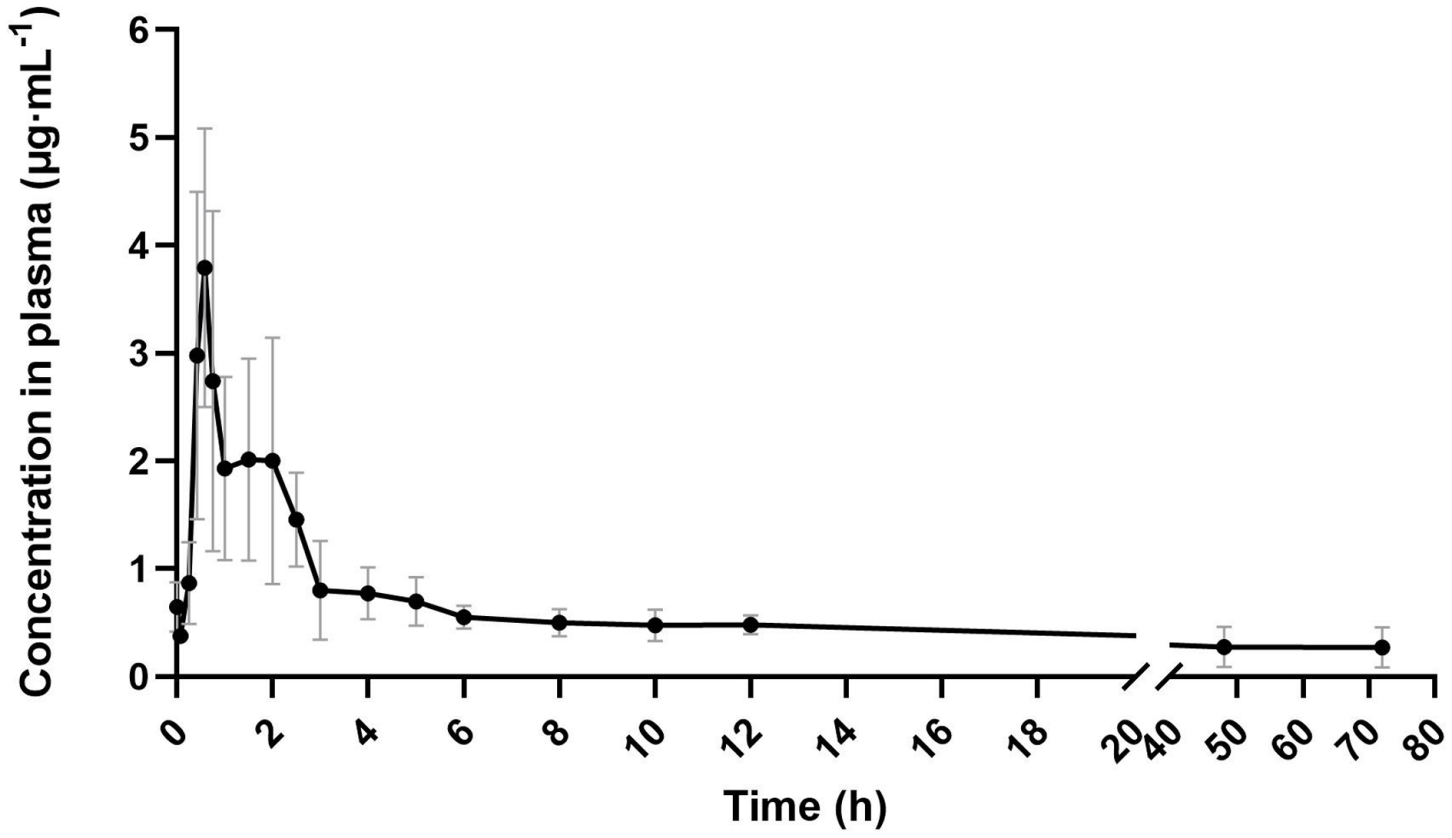
Concentration-time curve of neomycin in plasma in donkeys following a single intragastric administration of 30 mg·kg^−1^ BW, *n* = 5.

The plasma concentration-time profile of neomycin in donkeys following a single intragastric administration is presented in Figure 2, and the corresponding pharmacokinetic parameters are summarized in Table 4. The results indicated that the plasma concentration of neomycin increased over time, peaked at 0.85 ± 0.36 h (T_max_) with a C_max_ of 4.05 ± 1.99 μg·mL^−1^. Notably, neomycin remained detectable in plasma for up to 72 h post-administration. Analysis of the pharmacokinetic data revealed a slow elimination phase. The terminal elimination half-life (T_₁/₂λz_) was 32.14 ± 12.71 h, and the area under the concentration-time curve from time zero to infinity (AUC_₀–∞_) was 42.04 ± 15.51 μg·mL^−1^·h^−1^. The mean residence time (MRT) was 48.38 ± 22.78 h. These results collectively indicate that neomycin is rapidly absorbed into the systemic circulation of donkeys, but exhibits low peak concentrations and slow elimination.

**Table 4:** Pharmacokinetic parameters of neomycin in plasma of donkeys following a single intragastric administration of 30 mg·kg^−1^ BW, *n* = 5.

Items	Neomycin
λz (1·h^−1^)	0.03 ± 0.02
T_1/2λz_ (h)	32.14 ± 12.71
T_max_ (h)	0.85 ± 0.36
C_max_ (μg·mL^−1^)	4.05 ± 1.99
AUC_0-∞_ (μg·mL^−1^·h^−1^)	42.04 ± 15.51
MRT (h)	48.38 ± 22.78

λz, the first order rate constant associated with the terminal portion of the curve; T_1/2λz_, terminal half-life; AUC_0-∞_, area under curve; T_max_, time of maximum observed concentration; C_max_, maximum observed concentration; MRT, mean residence time.

### 3.3 Pharmacokinetic parameters of neomycin in the urine of donkeys

**Figure 3:**
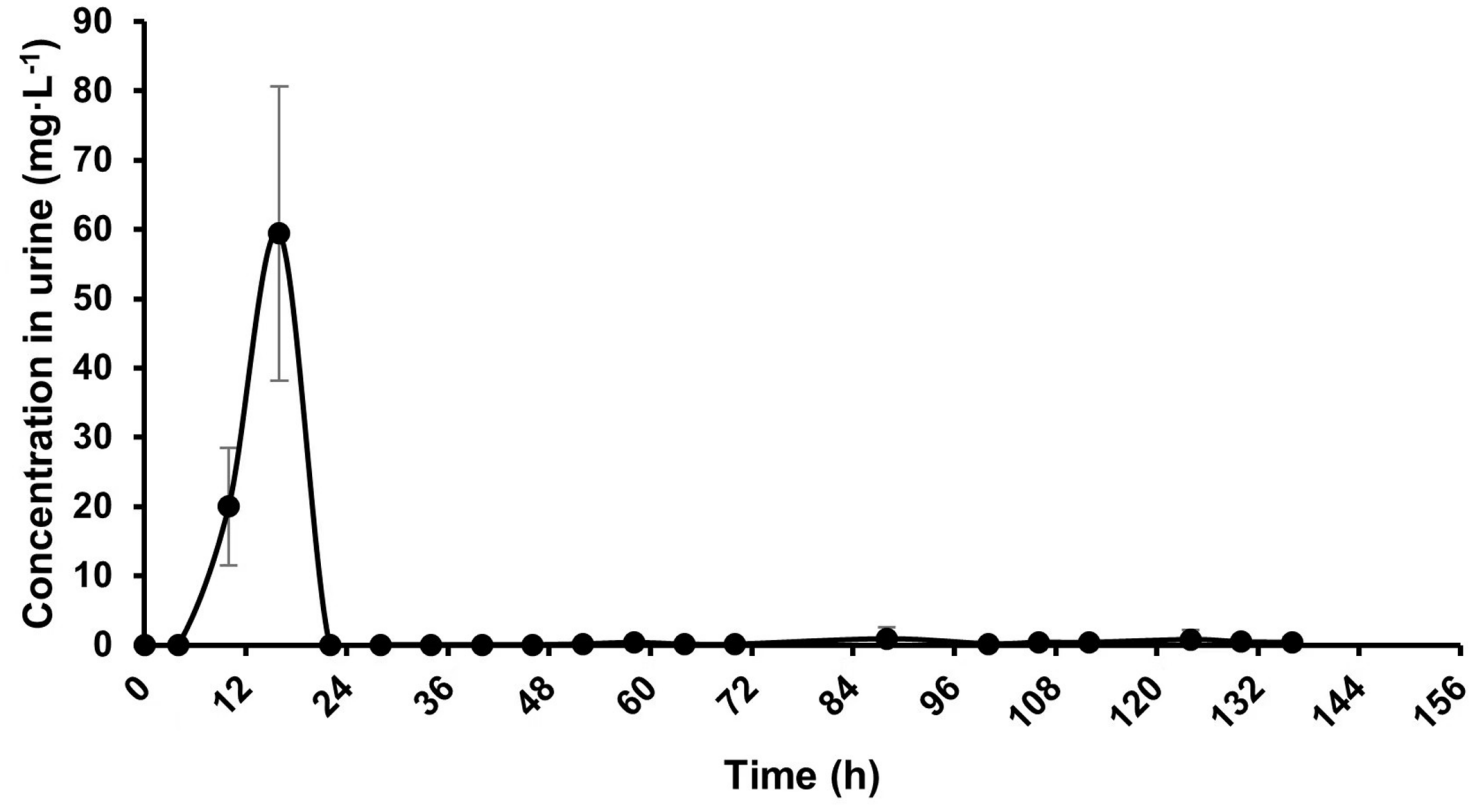
Concentration-time curve of neomycin in urine in donkeys following a single intragastric administration of 30 mg·kg^−1^ BW, *n* = 5.

Following a single intragastric administration of 30 mg·kg^−1^ BW neomycin in donkeys, the peak concentration in urine was observed at 16 h, with a C_max_ of 59.41 ± 21.24 μg·mL^−1^. Subsequently, the neomycin concentration in urine declined rapidly and became almost undetectable after 24 h (Figure 3). The urinary pharmacokinetic parameters of neomycin in donkeys are presented in Table 5. The results indicated that the maximum excretion rate of 7.25 ± 9.56 mg·h^−1^ was reached at 15.00 ± 2.83 h. The terminal half-life (T_₁/₂λz_) in urine was 44.90 ± 47.12 h. The amounts of neomycin cumulatively recovered in urine were 656.18 ± 545.81 mg. Neomycin combined were recovered in (15.08 ± 12.55)% of the total amount administered.

**Table 5:** Pharmacokinetic parameters of neomycin in urine of donkeys following a single intragastric administration of 30 mg·kg^−1^ BW, *n* = 5.

Items	Neomycin
λz (1·h^−1^)	0.04 ± 0.02
T_1/2λz_ (h)	44.90 ± 47.12
Time of maximum rate (h)	15.00 ± 2.83
Maximum excretion rate (mg·h^−1^)	7.25 ± 9.56
AURC_0-∞_ (μg·mL^−1^·h^−1^)	97.51 ± 114.24
Recovered amount (mg)	656.18 ± 545.81
Total recovered percent (%)	15.08 ± 12.55

λz, the first order rate constant associated with the terminal portion of the rate curve; T_1/2λz_, terminal half-life; C_max_, maximum observed concentration; AURC_0-∞_, area under rate curve.

### 3.4 Pharmacokinetic parameters of neomycin in feces of donkeys

**Figure 4:**
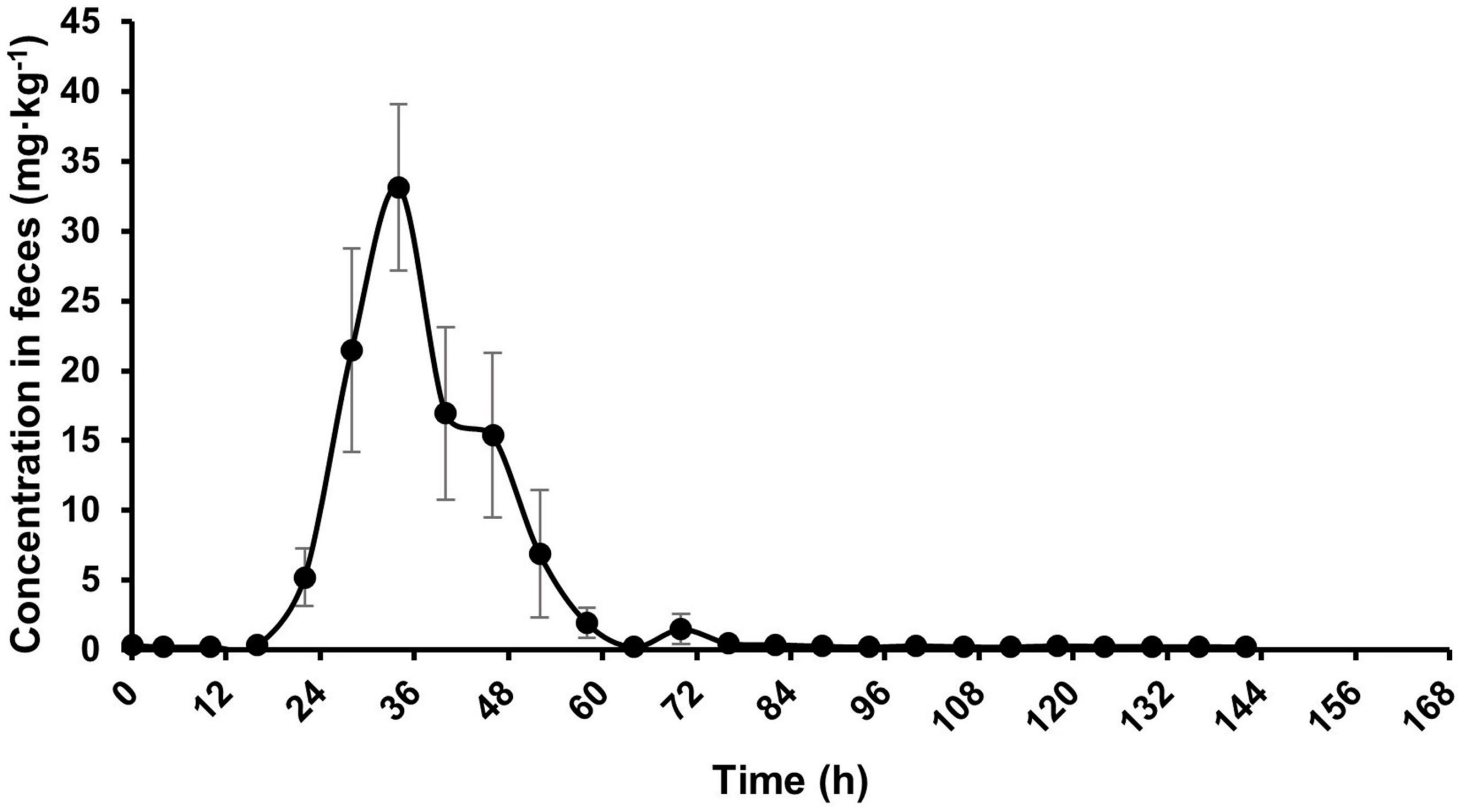
Concentration-time curve of neomycin in feces in donkeys following a single intragastric administration of 30 mg·kg^−1^ BW, *n* = 5.

After intragastric administration in donkeys, neomycin was first detected in feces at 18 h, and then increased to maximum levels around 36 h. The concentration of neomycin in the feces declined and fell below the limit of detection by 144 h post-administration (Figure 4). As shown in Table 6, the cumulative excretion of neomycin in feces was 3087.96 ± 91.93 mg, accounting for (70.99 ± 2.11)% of the administered dose following a single intragastric administration of 30 mg·kg^−1^ BW. These results indicate that neomycin is predominantly excreted via the fecal route.

**Table 6:** Pharmacokinetic Parameters of neomycin in feces in donkeys following a single intragastric administration of 30 mg·kg^−1^ BW, *n* = 5.

Items	Neomycin
Recovered amount (mg)	3087.96 ± 91.93
Total recovered percent (%)	70.99 ± 2.11

### 3.5 Pharmacokinetic/pharmacodynamic parameters for neomycin in donkeys

In the present study, after a single intragastric administration of 30 mg·kg^−1^ BW neomycin to donkeys, the plasma C_max_ was 4.05 ± 1.99 μg·mL^−1^, and the AUC_0-∞_ was 42.04 ± 15.51 μg·mL^−1^·h^−1^. Based on the pharmacokinetic/pharmacodynamic (PK/PD) targets for aminoglycosides (AUC₂₄/MIC ≥ 80 or C_max_/MIC ≥ 10), the calculated indices (Table 7) suggest that this dosing regimen could potentially inhibit pathogens with a MIC ≤ 0.5 μg·mL^−1^. When compared with historical MIC data for equine pathogens (Table 8) (34), this threshold indicates that the administered dose may be effective against *Klebsiella pneumoniae*, *Escherichia coli*, *Salmonella Typhimurium*, *Corynebacterium equi*, and *Streptococcus equi*. Efficacy against isolates with higher MICs is predicted to be limited.

**Table 7:** PK/PD parameters of neomycin in plasma in donkeys following a single intragastric administration of 30 mg·kg^−1^ BW, *n* = 5.

Item	Parameter	MICs (μg·mL^−1^)						
		0.03	0.06	0.12	0.25	0.50	1.00	2.00
Plasma	AUC/MICs	1401.33	700.67	350.33	168.16	83.91	42.04	21.02
	C_max_/MICs	135	67.5	33.75	16.2	8.08	4.05	2.03

AUC, area under curve; MICs, minimum inhibitory concentrations; C_max_, maximum observed concentration.

**Table 8:** Minimum inhibitory concentrations (MICs) of neomycin against equine pathogenic bacteria.

Pathogenic bacteria	MICs (μg·mL^−1^)
Gram-negative bacteria	
*Pseudomonas aeruginosa*	3.125–200
*Klebsiella pneurnoniae*	0.78–200
*Escherichia coli*	0.78–200
*Salmonella typhimurium*	0.78–12.5
*Hafnia alvei (Enterobacter ha fniaj)*	1.56–3.125
*Enterobacter cloaca*	3.125–6.25
*Proteus mirabilis*	12.5–200
Gram-positive bacteria	
*Corynebacterium equi*	0.78–6.25
*Staphylococcus aureus*	1.56–200
*Streptococcus equi*	0.78–6.25
*Streptococcus zooepidemicus*	3.125–12.5
*Streptococcus equisimiis*	1.56–6.25

MICs, minimum inhibitory concentrations. MICs data were reported by Baggot et al. (23).

## 4 Discussion

This study is the first to systematically elucidate the pharmacokinetic characteristics of neomycin in plasma, urine, and feces following intragastric administration in donkeys. Although our team has previously conducted a series of pharmacokinetic studies on other classes of antibiotics in donkeys, such as the fluoroquinolone enrofloxacin, the macrolide tilmicosin, and the amphenicol florfenicol (35–37), the pharmacokinetic behavior of neomycin, as a representative aminoglycoside antibiotic, remains unreported in this specific species. Therefore, the primary necessity of this research lies in addressing the lack of species-specific pharmacokinetic data for neomycin in donkeys, which are economically significant animals. This work provides direct evidence to support its rational clinical application. Regarding innovation, this study represents the first comprehensive elucidation of the pharmacokinetic profile of neomycin in plasma, urine, and feces of donkeys, and clearly demonstrates that its elimination occurs predominantly via fecal excretion.

Neomycin sulfate is a widely used aminoglycoside antibiotic in veterinary medicine for treating severe bacterial infections, including pullorum disease in chickens, white scours in piglets, endometritis and mastitis in dairy cows (34). Improper use of the drug, failure to observe the withdrawal period, or slaughtering animals during treatment can lead to drug residues in tissues, posing risks of bacterial resistance development and potentially reducing the drug’s effectiveness as a human therapeutic agent (31). Persistent aminoglycoside residues in animal-derived foods may pose allergenic and toxic risks to consumers, such as ototoxicity, nephrotoxicity, teratogenic effects, and rare neuromuscular blockade or hypersensitivity reactions (38, 39). Therefore, caution or dose reduction is advised for pregnant jennies, foals, and other susceptible animals. Consequently, conducting pharmacokinetic studies in specific species is essential, as it not only relates to the economic efficiency of livestock production but also has significant implications for human health.

The present study demonstrates that following intragastric administration of neomycin to donkeys, the drug is rapidly absorbed yet exhibits a remarkably prolonged elimination phase. Pharmacokinetic studies on neomycin in equine species are limited. In a previous investigation conducted in horses, administration of neomycin at 10 mg·kg^−1^ BW by intravenous and intramuscular routes resulted in serum half-lives of 2.10 ± 0.97 h and 2.58 ± 0.69 h, respectively. After intramuscular injection, a peak serum concentration of 23.2 ± 10.2 μg·mL^−1^ was attained within 30 min, with a mean serum level of 2.8 ± 0.8 μg·mL^−1^ observed at 8 h post-administration. These data suggest that neomycin is absorbed very rapidly and distributes well after injection (23). The considerable differences between the aforementioned equine studies and the results of the present trial are likely primarily attributable to the different routes of administration. The low oral bioavailability of neomycin, due to its poor absorption from the gastrointestinal tract, limits its systemic exposure, which is reflected in the relatively low peak plasma concentration observed in this study compared to the serum levels achieved after intramuscular injection in horses. However, the route of administration may not be the sole determining factor. Interspecies physiological and metabolic differences between donkeys and horses likely also play a significant role. This is supported by the observation that even when considering absorption efficiency, the peak serum concentration in horses was substantially higher than that in donkeys, suggesting species-dependent absorption characteristics. It is well-documented that substantial variations exist in physiological and metabolic profiles across species, including disparities in body water compartments and the activity of drug-metabolizing enzymes such as the cytochrome P450 (CYP) enzyme system (8, 9). These factors can profoundly influence drug absorption, distribution, and elimination. Therefore, the prolonged elimination half-life observed in donkeys is a critical finding. It is noteworthy that neomycin exhibits this prolonged half-life in donkey plasma, indicating a potential risk of nephrotoxicity with repeated administration, despite its limited systemic absorption.

Following intravenous administration in Swine (15 mg·kg^−1^ BW), neomycin exhibited a relatively rapid elimination (T_₁/₂λz_ = 5.87 ± 1.12 h; MRT = 6.07 ± 0.49 h) and an area under the AUC_₀–∞_ = 76.14 ± 3.52 μg·h·mL^−1^. In contrast, after intragastric administration of the same dose, the pharmacokinetic profile changed markedly, showing prolonged elimination (T_₁/₂λz_ = 12.43 ± 7.63 h), delayed absorption (T_max_ = 1.92 ± 0.97 h), and drastically reduced bioavailability (*F* = 4.84% ± 0.03) (24). Similarly, in ruminants such as sheep and cattle, neomycin also demonstrates distinct kinetic behaviors. In sheep, the drug is rapidly distributed (T_₁/₂*α*_ = 3.16 min) and eliminated (T_₁/₂*β*_ = 1.98 h) with a large volume of distribution (304.69 mL·kg^−1^) (25). Calves showed variable results depending on the route: after intramuscular administration, bioavailability was notably high (*F* = 127 ± 35.2%), whereas oral administration led to minimal systemic absorption (*F* = 0.45 ± 0.45%) (27) (Table 9). These interspecies differences in pharmacokinetics are likely attributable to distinct digestive physiologies (e.g., ruminant versus monogastric systems) and associated dietary patterns, both of which critically influence gastrointestinal transit and drug absorption. Based on the experimental data, approximately 15.08% of the administered neomycin was recovered in urine, indicating that renal excretion serves as a significant elimination pathway. This finding suggests that neomycin may also be suitable for the treatment of urinary system diseases caused by susceptible pathogens, which is consistent with commonly used clinical treatment protocols in veterinary practice (40). Similar to the published studies mentioned above, this exploratory pharmacokinetic investigation, conducted in a large animal model, employed a relatively limited sample size (*n* = 5). This sample size is consistent with those in comparable studies and provides robust preliminary estimates of key pharmacokinetic parameters.

**Table 9:** Pharmacokinetic study of neomycin in different animals.

Species	Route of administration	Dose (mg·kg^−1^·BW^−1^)	Elimination half-life (h)	C_max_ (μg·mL^−1^)	T_max_ (h)	AUC (μg·h·mL^−1^)	References
Horse	Intravenous	10	2.10	–	–	–	Baggot et al. (23)
	Intramuscular	10	2.58	23.20	0.50	–	
Swine	Oral	15	12.43	0.11	1.92	1.55	Liu et al. (24)
	Intravenous	10	5.87	15.80	0.30	80.31	
Sheep	Intravenous	10	1.98	–	–	94.54	Errecalde et al. (25)
	Intramuscular	10	2.68	17.63	1.33	70.29	
	Subcutaneous	10	2.82	18.66	1.0	80.18	
Cattle	Intravenous	12	2.78	–	–	0.56	Black et al. (44)
	Intravenous	12	37.48	–	–	51.0	Pedersoli et al. (27)
	Intramuscular	24	11.50	31.7	1.38	126.1	
	Oral	95	–	0.26	2.6	1.85	

C_max_, maximum observed concentration; T_max_, time of maximum observed concentration; AUC, area under curve.

The high recovery of the administered neomycin dose in feces (70.99%) unequivocally identifies fecal excretion as the predominant elimination pathway following intragastric administration in donkeys. This fraction predominantly represents the unabsorbed drug, which exerts a direct local antibacterial effect within the intestinal lumen, thereby providing a robust pharmacokinetic rationale for its efficacy in treating bacterial enteritis. The observed fecal excretion pattern and percentage are consistent with the drug’s inherent poor lipid solubility and minimal gastrointestinal absorption reported in other species (41). Notably, the substantial amount of active drug excreted in feces underscores the importance of prudent environmental stewardship in farm settings. Appropriate management of animal waste is crucial to mitigate the potential selection for resistant bacteria in the environment and to reduce the risk of ecotoxicological impact.

Aminoglycosides such as neomycin are considered concentration-dependent bactericidal agents. For this class of drugs, the ratio of peak plasma concentration to minimum inhibitory concentration (C_max_/MIC) is the primary predictor of efficacy, while the ratio of the area under the concentration-time curve to MIC (AUC/MIC) is associated with therapeutic effectiveness and the prevention of resistance development. In human medicine, an AUC_24_/MIC ≥ 80 and C_max_/MIC ≥ 10 are recognized as optimal targets for achieving therapeutic efficacy against bacterial infections (42, 43). However, it has been reported that in immunocompetent animals, an AUC_24_/MIC ≥ 40 may be sufficient for the treatment of certain infections (41). Therefore, when treating donkeys infected with pathogens that have an MIC > 0.25 μg·mL^−1^, neomycin may not be a suitable choice. The MICs of several bacteria isolated from equines are compiled based on previous studies (38). In conjunction with the pharmacokinetic results from the present study, the dose of neomycin in our study was predicted to be effective for pathogenic bacteria with *Klebsiella pneumoniae*, *Escherichia coli*, *Salmonella Typhimurium*, *Corynebacterium equi*, and *Streptococcus equi*. In contrast, the efficacy against other pathogens appears to be limited. However, it is important to note that the referenced MIC data were sourced from a report published in 1985, which is now nearly four decades old. Temporal changes in bacterial susceptibility profiles, potentially influenced by decades of antibiotic use, remain uncharacterized. Consequently, updated MIC determinations for contemporary equine-derived pathogens are urgently required to accurately assess the current clinical relevance of neomycin. Crucially, this PK/PD assessment pertains only to the systemically absorbed fraction. The primary mode of action for intragastric neomycin is likely direct contact at the intestinal site of infection, where concentrations vastly exceed systemic levels and any relevant MIC. Nevertheless, the prolonged half-life (~32 h) of the absorbed fraction indicates that if therapeutic systemic concentrations are achieved, they would be sustained, but it also significantly raises the risk of cumulative nephrotoxicity with repeated dosing, narrowing the drug’s safety margin. Furthermore, although donkeys and horses belong to the same genus, differences in physiological characteristics and drug metabolism exist between the two species. Therefore, whether the MICs established for equine pathogens are directly applicable to donkeys requires further investigation.

## 5 Conclusion

Following a single intragastric dose of 30 mg·kg^−1^ BW in donkeys, neomycin was rapidly but poorly absorbed, with a low peak plasma concentration (C_max_ = 4.05 ± 1.99 μg·mL^−1^; T_max_ = 0.85 ± 0.36 h). The absorbed fraction exhibited an unexpectedly prolonged elimination half-life (T_₁/₂λz_ = 32.14 ± 12.71 h), indicating a significant risk of systemic accumulation. Consequently, neomycin was predominantly excreted unchanged in feces (70.99% of the dose), supporting its primary role as a local intestinal antimicrobial. Urinary excretion accounted for only 15.08% of the administered dose. It is predicted to be suitable for treating intestinal infections, but caution is warranted regarding the risk of accumulation with prolonged use. These results not only clarify the drug’s kinetic profile but also provide essential pharmacokinetic evidence to support the advancement of precision medicine in donkey internal medicine.

## Data Availability

The original contributions presented in the study are included in the article/supplementary material, further inquiries can be directed to the corresponding authors.
